# Thiamine as a metabolic resuscitator in septic shock: a randomized, double-blind, placebo-controlled, pilot trial

**DOI:** 10.1186/cc14472

**Published:** 2015-03-16

**Authors:** M Donnino, LW Andersen, M Chase, KM Berg, TA Giberson, H Smithline, M Tidswell, R Wolfe, M Cocchi

**Affiliations:** 1 Beth Israel Deaconess Medical Center, Boston, MA, USA; 2 Baystate Medical Center, Springfield, MA, USA

## Introduction

The objective was to determine whether the administration of thiamine mitigates elevated lactate levels in patients with septic shock. Thiamine is essential for aerobic metabolism and we have found that thiamine levels are low and inversely correlated with lactate levels in patients with sepsis.

## Methods

We performed a randomized, double-blind, placebo-controlled, two-center trial from January 2010 to October 2014. We enrolled patients with septic shock, elevated lactate (≥3 mmol/l) and no obvious competing cause of lactate elevation. Patients received thiamine 200 mg or placebo i.v. twice/day for 7 days. The primary outcome was lactate levels at 24 hours. Secondary outcomes included the SOFA score at 24 hours and mortality. Lactate levels at 24 hours were compared between groups using the Wilcoxon rank-sum test and categorical variables were compared using the Fisher's exact test. Lactate values at 24 hours, for those who died before 24 hours, were imputed according to a predefined plan. We performed a preplanned analysis in those with baseline thiamine deficiency (≤7 nmol/l).

## Results

We enrolled 88 patients; 43 received thiamine and 45 placebo. Baseline characteristics were similar between groups. We found no overall statistical significant difference in 24-hour lactate levels between thiamine and placebo groups (2.5 (IQR: 1.5 to 3.4) vs. 2.6 (IQR: 1.6 to 5.1), *P *= 0.40). Fewer patients in the thiamine group had lactate levels >4 mmol/l at 24 hours (21% vs. 38%, *P *= 0.10) and this was statistically significant if only evaluating survivors at 24 hours (7% vs. 33%, *P *= 0.03), although our preplanned analysis was to impute data. We found no difference in 24-hour SOFA score or mortality. A total of 28 (35%) patients were thiamine deficient. Of the deficient patients, those receiving thiamine had statistically significant lower lactate levels at 24 hours (2.1 (IQR: 1.4 to 2.5) vs. 3.1 (IQR: 1.9 to 8.3), *P *= 0.03) and more patients in the placebo group had a lactate >4 mmol/l (38% vs. 7%, *P *= 0.07). Mortality in the thiamine and placebo groups was 13% and 46%, respectively (*P *= 0.10).

## Conclusion

Thiamine deficiency is prevalent in septic shock. Thiamine did not decrease overall median lactate levels at 24 hours. In the patients with thiamine deficiency, there were statistically significant lower lactate levels at 24 hours in the thiamine group and a large, although nonsignificant, difference in mortality.

